# Multicenter evaluation of the Waveband system for automated sleep assessment in patients with insomnia symptoms

**DOI:** 10.1093/sleep/zsag069

**Published:** 2026-03-28

**Authors:** Silvia Frati Savietto, Antoine Guillot, Mason Harris, Jay Pathmanathan, Alexander M Chan, M Brandon Westover, Derek Hill, Valérie Bertaina-Anglade, Geoffrey Viardot, Pierrick Arnal, Jacob Donoghue

**Affiliations:** Beacon Biosignals, Boston, MA, USA; Beacon Biosignals, Boston, MA, USA; Beacon Biosignals, Boston, MA, USA; Beacon Biosignals, Boston, MA, USA; Beacon Biosignals, Boston, MA, USA; Beacon Biosignals, Boston, MA, USA; Panoramic Digital Health, Grenoble, France; Biotrial Neurosciences, Rennes, France; Biotrial Neurosciences, Rennes, France; Dreem SAS, Paris, France; Beacon Biosignals, Boston, MA, USA

**Keywords:** sleep assessment, Dreem3S, automatic sleep staging, polysomnography, performance, dry-EEG

## Abstract

**Study Objectives:**

This study (Octave-3) aimed to validate the performance of the Waveband dry-electroencephalography sensor device for automatic sleep staging and sleep parameter estimation as compared to gold-standard in-lab polysomnography (PSG).

**Methods:**

Forty-five participants were enrolled, and 38 completed simultaneous PSG and Waveband recordings. PSG data were scored by six human technologists, while Waveband data were scored algorithmically. Agreement between sleep staging results and derived sleep parameters (total sleep time (TST), sleep efficiency (SE), sleep onset latency (SOL), latency to 10 minutes of persistent sleep (LPS), wake after sleep onset (WASO), and time spent in each sleep stage) was measured using the Intraclass Correlation Coefficient (ICC) and overall agreement (OA). Waveband OA was compared to each human rater using the leave-one-out consensus of the remaining five human experts.

**Results:**

Average OA between Waveband vs the leave one out consensuses was 87.3+/-5.4 per cent, equivalent to the average OA for individual human experts of 85.9+/-7.6 per cent (*p* > .1). Waveband and humans had better OA over the second half of the night, but Waveband had superior OA. ICCs for TST, SE, LPS, and WASO exceeded 0.9, indicating excellent agreement between automated Waveband and human PSG scoring. Lower agreement was found for time spent in N1, N3, and Rapid Eye Movement Sleep (REM), with ICCs ranging from 0.65 to 0.73.

**Conclusions:**

Waveband provides accurate sleep staging and estimation of TST, SE, SOL, LPS, and WASO, with comparable performance to human expert staging of PSG. Its reduced form-factor and good performance should make it a valuable tool for automated assessment of sleep in patients with disturbed sleep.

**Clinical Trial Information**

Trial name: Octave-3.

URL: https://www.clinicaltrials.gov/study/NCT05438017

Registration: NCT05438017.

## Graphical Abstract

**Graphical Abstract:**
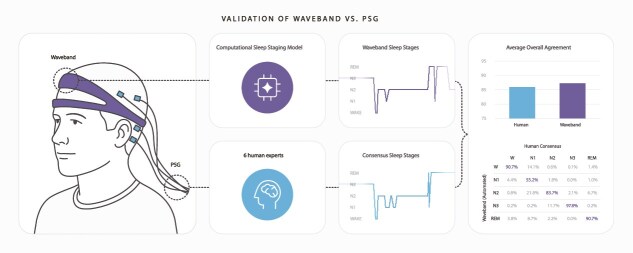

Statement of Significance This study demonstrates the high accuracy of the Waveband system (with a simplified interface designed for patient self-operability for ambulatory sleep monitoring and automated machine learning sleep staging). Waveband performance was comparable to the consensus of 6 human experts scoring in-lab clinical polysomnography (PSG). Waveband is a non-invasive and easy-to-use headband (operable directly by patients with disturbed sleep) that allows for multiple nights of data collection in the home environment. Waveband offers significant advantages over traditional PSG monitoring, including reduced burden on patients, automated sleep staging output, and the ability to capture sleep in the home environment over multiple nights. The simplicity of Waveband operation makes it an alternative to actigraphy, allowing for direct measurement of cerebral sleep state. Waveband is aimed at broadening the ability to investigate the neuropsychiatric underpinnings of sleep pathology and improve diagnosis and management of common sleep disorders.

## Introduction

Sleep disorders are a common feature of many neuropsychiatric conditions, including neurodegenerative diseases [1–5], epilepsies [6–9] narcolepsy, insomnia, depression [10–14], and Post Traumatic Stress Disorder (PTSD) [15]. For many of these conditions, sleep pathology is an underrecognized but treatable comorbidity contributing to disease severity. In some cases, sleep pathology may be a fundamental biomarker of the disease itself.

The gold standard for sleep measurement, polysomnography (PSG), is an excellent measure of sleep and tool to investigate a variety of root cause disturbances. However, PSG is expensive in terms of cost, time of skilled technologists, and patient burden. Use of PSG in the clinical setting is limited to a few indications, and longitudinal PSG to monitor sleep changes over days to weeks is impossible. Actigraphy, which is often accepted as a sleep staging endpoint, has shown poor specificity for differentiating sleep from motionless wakefulness, especially in patients with fragmented sleep [16]. Therefore, relying on actigraphy may result in overestimation of total sleep time (TST) and underestimation of wake after sleep onset (WASO) time [16, 17]. Consequently, electroencephalography (EEG)-based sleep monitoring may provide valuable data in neurological and psychiatric conditions impacting sleep, but it is not generally available outside a few conditions labeled as primary sleep or epileptic disorders.

Advances in microelectronics allow for novel and simplified EEG-based systems as a promising non-invasive approach to longitudinal monitoring of sleep in the home environment. Such systems can provide measures of sleep architecture (both macroscopic elements, such as sleep stage by 30-second epochs, and sleep microarchitecture, such as sleep spindle metrics, K-complexes, and spectral power analyses) and quality [18].

Reduced-montage, easy-to-wear EEG devices include headbands [19, 20] and devices placed in or around the ear [21, 22]. They are designed to be worn at home and typically do not require expert supervision, making them an attractive option for clinicians. However, these devices do not use standard PSG electrode montages, may be prone to a variety of errors and artifacts due to lack of technologist placement, and rely on automated methods given the non-standardized data measurement. Therefore, results from these systems must be objectively validated prior to acceptance [17]. EEG headbands have been compared to PSG, with overall agreement (OA) to automated staging in the 80 per cent range [19, 23] and ICC values for sleep staging and sleep metrics in the 0.8 range—demonstrating good correlation to PSG-based staging when using frontal EEG.

The Waveband headband (originally branded as Dreem 3) is a self-applied (patient-operated) dry-EEG system intended to monitor sleep for multiple nights in the home setting. Waveband collects EEG from dry-EEG sensors located frontally and occipitally, unlike prior EEG bands that focused on frontal leads. Waveband also uses 3D accelerometry data during operation, with sensor data being wirelessly transferred to a secure server for algorithmic analyses after data collection. Outputs include automated sleep staging for each 30-second epoch. This output is produced using an algorithm that analyzes raw data from the headband EEG and accelerometer sensors to provide automatic sleep staging according to the American Academy of Sleep Medicine (AASM) classification rules [24]. The neural network architecture is a hierarchical sequence-to-sequence model inspired by Guillot *et al.* [25].

The purpose of this study was to validate the sleep staging outputs and data quality of the Waveband system by comparing results from the Waveband system to simultaneously recorded in-lab PSG. In this study, participants with chronic insomnia were selected as this is a common neuropsychiatric condition that is not typically evaluated with in-lab PSG but reflects the broader range of sleep disorders that might benefit from quantitative sleep study. Sleep technologists placed both the Waveband system and standard PSG as per AASM guidelines. Data from the clinical PSGs were scored by three independent human sleep staging experts, and the consensus hypnogram was compared to the output from the algorithms of the Waveband headband. Results from the present study were used as part of the United States Food and Drug Administration (FDA) 510 k clearance of the Waveband system (K223539). A separate study (Livie-1) examined use of the Waveband system by participants (without trained technologist assistance) in the home setting.

## Materials and Methods

### Subjects

Forty-five participants were recruited from two sites within Biotrial’s clinical units in Newark, NJ, United States, and Rennes, France. This sample ensured sufficient power for both non-inferiority and reliability endpoints, accounting for 10 per cent attrition. A sample of 37 provides 80 per cent power (α = 0.05) to demonstrate non-inferiority for wake detection (margin = 0.667, expected mean = 0.73, SD = 0.15) and 36 ensures adequate precision to estimate an ICC of 0.80 with 95 per cent confidence. The inclusion criteria required adults aged 22–70 years to report self-perceived chronic insomnia symptoms, assessed using the International Classification of Sleep Disorders, third edition (ICSD-3) criteria and confirmed by a clinician. Exclusion criteria included pregnancy or breastfeeding, a Body Mass Index (BMI) equal to or greater than 40, and use of alcohol, drugs, or medications that could induce somnolence or wakefulness or interfere with sleep recording. Participants with a history of obstructive sleep apnea (OSA), REM sleep disorder, narcolepsy, hypersomnia, or significant breathing difficulties due to underlying conditions, as well as those diagnosed with Central Nervous System (CNS) disease or unstable medical conditions, were excluded. Participants who were subsequently found to have moderate or severe OSA (or other incidental findings on PSG) were also excluded from the study. Of the 45 enrolled participants, 44 participants completed one night of recording (one developed a tooth abscess after enrollment). Two trained human experts [one clinical operations neuroscientist experienced in evaluating Waveband data and one registered PSG technologist, Registered Polysomnographic Technologists (RPSGT)] independently assessed the quality of both PSG and Waveband raw data and deemed studies as either usable or unusable by visual inspection. A study was excluded if both experts deemed it unusable. Note that the Waveband data are assessed for low quality (both at startup and after recording ends), but this feature was not implemented for this study and will be evaluated separately.

### Assessments

Potential participants first underwent a brief phone screening with the Clinical Research Organization's screening staff, followed by an in-person screening at Biotrial’s clinical unit, led by the study staff under supervision of the principal investigator. On-site, participants provided demographic and medical history information, underwent a physical examination by the site staff, and underwent Covid testing and urine testing for drugs/alcohol to confirm eligibility. Participants were also screened for chronic insomnia based on ICSD-3 criteria by site staff (presence of a sleep disturbance including difficulty initiating or maintaining sleep causing dissatisfaction with sleep, daytime fatigue or neurocognitive consequence of impaired sleep, sufficient time for sleep without other known cause of sleep disturbance, sleep difficulties at least 3 times per week, and sleep difficulties present for at least 3 months). However, participants did not answer surveys aimed at characterizing the severity of their insomnia symptoms. The sleep clinic was required to maintain source documentation in accordance with Good Clinical Practice (GCP) guidelines and EN ISO 14155:2020 to substantiate all data collected.

Enrolled subjects were asked to arrive at the sleep laboratory around 8 pm. All PSG electrodes were placed on the subject’s head as per normal practice, and then the Waveband was positioned so that both systems could operate simultaneously. Participants were allowed to engage in quiet activities (eating, reading, film watching, chatting) before sleeping. The beginning and end of PSG and Waveband data collection periods were set based on participants’ self-selected lights-off and lights-on times to avoid interfering with their sleep habits. PSG and Waveband recordings were synchronized a posteriori by resampling Waveband data to the same timestamps as the PSG data. Following the sleep study, both devices were removed, and participants were debriefed and interviewed to identify any adverse events. The study was approved by an independent ethics committee (Advarra IRB) in the United States, and the Committees of Protection of Persons (CPP), declared to the French National Agency for Medicines and Health Products Safety, and carried out in compliance with the General Data Protection Regulation (EU GDPR), Health Insurance Portability and Accountability Act (HIPAA), GCP guidelines, International Conference on Harmonization (ICH) standards, and principles of the Declaration of Helsinki of 1964, as revised in 2013.

### Polysomnography

All participants underwent a full-night PSG recording in a sleep laboratory using a Natus portable EEG/PSG System. The PSG montage included EEG from the F3, F4, C4, C3, O1, and O2 scalp locations; bilateral electrooculography (EOG); chin electromyography (EMG); electrocardiography (EKG); and two respiratory belts to quantify movements of the chest and abdomen to estimate respiratory rate. No adaptations were made to the PSG (the Waveband was placed after the PSG electrodes, and positioned such that the Waveband occipital leads were just below the PSG occipital leads).

### Waveband

**Figure 1:**
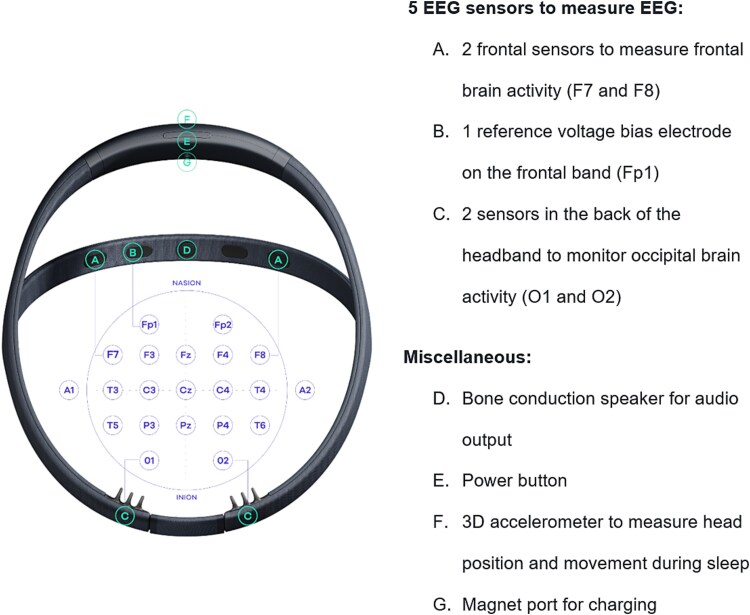
The Waveband EEG headband and its sensors.

The Waveband device, manufactured by Beacon Biosignals, is a headband that utilizes dry-EEG electrode technology, containing four electrodes (O1, O2, F7, F8; locations A and C in Figure 1) sampled at 250 Hz, with a voltage bias reference at Fp1 (location B in Figure 1), along with a 3D accelerometer sampled at 50 Hz. The headband is depicted in Figure 1.

The EEG signal is captured in bipolar pairs from four electrodes, two located in the frontal region (roughly F7/F8 position) and two located at the back of the head (roughly O1/O2 position). An embedded 3D accelerometer on the headband allows measurements of head movement, which are used to identify movements and respiration-like patterns during sleep. Respiratory-like traces are only available as raw signals, but no respiratory outputs are provided after processing.

**Figure 2:**
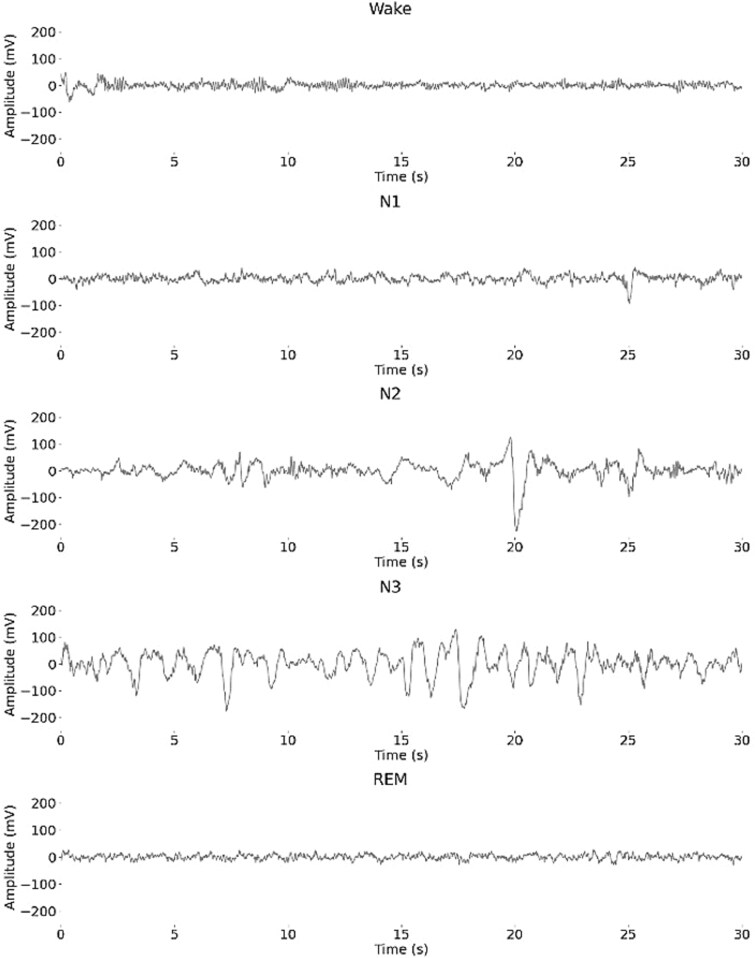
Representative EEG signals from the frontal-occipital derivations captured by the Waveband are displayed. During N2, spindles and K-complexes are easily identifiable, while slow waves are visible during N3. The wake sample displays high alpha power.

The EEG data from five derivations (F7-O1, F7-O2, F8-O1, F8-O2, and F7-F8) are bandpass filtered between 0.4 and 18 Hz, then notch filtered at 50, 60, and 62.5 Hz to eliminate potential powerline interference. The accelerometer data are filtered between 0.1 and 0.5 Hz to extract respiration-like patterns. After data acquisition and transfer to a secure cloud server, the EEG and accelerometer signals are analyzed by a neural network to determine sleep stages in 30-second intervals. The neural network’s architecture is based on RobustSleepNet [25] and SimpleSleepNet [26]. The algorithm was trained and then locked prior to the study, with no data from this study, any of its participants, or any of the study locations being used for training or validation. Figure 2 illustrates an EEG data sample captured by Waveband. Two human experts evaluated the quality of the EEG data before inclusion in the analysis dataset (as described above).

### Alignment of data from the Waveband and PSG

Since Waveband and PSG use different clock systems, their data had to be aligned to ensure that the sleep epochs from both devices corresponded accurately. To improve and check this alignment, a biocalibration procedure was performed in which subjects were instructed to blink five times at one-second intervals before and after lights off time. Alignment of the data from both devices was carried out automatically and then verified manually using the biocalibration signal above.

### Scoring of the PSG data

Due to the interindividual variability in sleep scoring among different experts [18], the original study utilized PSG-derived sleep stage labels generated from the consensus opinion of 3 human experts as the gold standard reference point. PSG data were scored in 30-second epochs according to the AASM manual for scoring sleep and associated events (V.2.4) [24] by three RPSGTs (from Biotrial Neurosciences) who were blinded to the Waveband data, plus reviews from 3 additional independent and blinded RPSGTs for post hoc agreement analyses outlined below. The following sleep stages were identified: wake, non-rapid eye movement (NREM) stages N1, N2, and N3, and rapid eye movement (REM) sleep. These scores were used to generate a consensus hypnogram. To establish this consensus for the original three scorers, each of the three hypnograms was compared with one another to determine the most accurate sleep staging using the majority method of comparison (2/3 majority rule). Scorers were ranked based on their level of agreement with the hypnograms of the other two scorers, with the highest-ranked scorer being considered the top scorer. For segments without consensus (all three humans used different labels), the top scorer’s stage label was used.

Post-hoc analyses were conducted after submission of the original data packet to the FDA and were not part of the original analysis plan. These analyses were conducted to obtain a better assessment of human agreement (without need to determine top scorer for tie breaking) and to compare Waveband and individual human performance as compared to a human consensus score that was independent of the human comparator. Therefore, we obtained scoring from an additional three human RPSGTs, for a total of six human reviewers per study. OA was calculated using the aggregate of 5 human reviewers, leaving one out (for a total of 6 OA scores: scorer 1 vs scorers 2–6, scorer 2 vs scorer 1 and 3–6, etc.), which we have labeled as the leave-one-out-consensus. In these analyses, consensus was the majority opinion, excluding any ties (e.g. if there were two votes for N1 and 2 votes for N2, then there was no consensus, but if there were two votes for N1 and 1 vote for W, N2, and REM, then the consensus would be N1). In this way, Waveband performance could be compared to individual human performance without the human being part of the gold standard. Paired two-tailed *t*-tests were used to determine the statistical significance of differences between OA for Waveband and OA for each human against the leave-one-out consensus. *p*-values were Bonferroni corrected for the number of comparisons.

### Pre-specified statistical analysis plan

The statistical analysis plan was established before the study and carried out by an independent statistician who remained blinded to the data collection. The primary success criterion was based on OA on the five sleep stages between Waveband and the consensus of the three PSG scorers (post hoc analyses using six human reviewers were not included in the original plan). This criterion was considered met if the lower bound of the 95 per cent bootstrapped confidence interval (CI) on the OA was above 80 per cent. Bootstrapping was conducted at a subject level.

Additionally, the 5 × 5 confusion matrix between Waveband and the consensus of the three scorers was computed over all scored epochs to provide supportive information. The positive and negative percent agreements for each sleep stage were also computed along with bootstrapped 95 per cent CIs. Bootstrapping for each sleep stage was also performed at a subject level.

To assess validity of the sleep parameter estimation by the Waveband, one-way random effects Intraclass Correlation Coefficients (ICCs) [27] and Bland–Altman plots were computed for each of the following sleep parameters (as defined in Table 1): total sleep time (TST), sleep onset latency (SOL), sleep efficiency (SE), LPS, WASO, and time spent in N1, N2, N3, and REM.

**Table 1:** Definition of the sleep metrics reported in the study

Parameter	Definition	Unit
TST	Sum of the time spent in N1, N2, N3, REM	min
SE	Ratio between the TST and the time in bed (time between light off and light on)	%
Time in N1, N2, N3, REM	Time spent in each of the sleep stages	min
Latency to persistent sleep (LPS)	LPS: time between light off and the beginning of the first continuous 20 epochs (i.e. 10 minutes) scored as non-awake, i.e., epochs scored as either N1, N2, N3, or REM	min
SOL	Time between light off and the first epoch of sleep (N1, N2, N3, or REM).	min
WASO	Wake duration between sleep onset and sleep offset.	min

## Results

### Demographics

Forty-four participants completed the study. Six nights were excluded—one due to diagnosis of moderate or severe OSA (2.3 per cent), one due to a failed PSG recording (2.3 per cent), one due to poor fit of the Waveband along with the PSG setup (poor fitment noted at the time of recording) (2.3 per cent), and three due to low-quality Waveband recordings on visual inspection (6.8 per cent). The low-quality Waveband recordings were due to excessive artifact including high-frequency noise and electrode pop, which is most frequently encountered due to loose contact, due to improper sizing (Waveband is too loose, and requires removal of an adjustable spacer). Ultimately, there were 38 evaluable recordings (86.3 per cent), out of which 23 (60.5 per cent) were from the United States site, and 15 (39.5 per cent) were from the European site. The included subjects had a mean age of 35.8 years ±11.6 (ranging from 23 to 66) and a median age of 32; 19 (50 per cent) were males, and 19 (50 per cent) were females. Data on race and ethnicity were collected by the United States site only. Out of the 29 subjects who completed the study at the United States site, 55 per cent (16/29) self-designated themselves as Black African American, 35 per cent (10/29) as White, and 10 per cent (3/29) as Asian. In compliance with French law, the race of subjects from the European sites was not collected. Demographics are summarized in Table 2.

**Table 2:** Demographics of the evaluable population of the study

	Mean ± SD	Min, Max
Female	19	NA
Male	19	
Black African American	14	NA
White	8	
Asian	1	
Not disclosed	15	
Age	35.8 ± 11.6	23; 66
BMI	28.2 ± 5.0	17.8; 37.8

### Sleep staging performance

**Figure 3:**
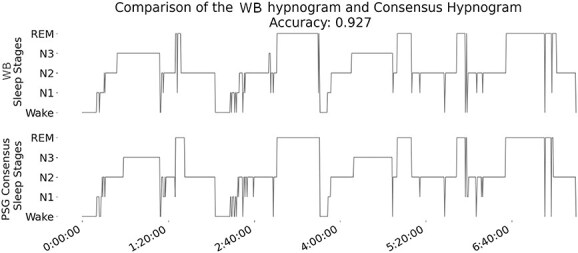
Comparison of the hypnogram from the Waveband (WB) and PSG consensus on a selected recording. The Waveband was able to detect sleep stages with very good agreement with the PSG on this recording. Short and long awakenings during the night are accurately detected.

Comparison between Waveband and the consensus hypnogram of the three expert scorers was carried out on the 38 recordings of the evaluable set. An example is provided in Figure 3. The set contained 36 474 scored epochs and 27 unscored epochs, with an average of 960 epochs per recording. The shortest and longest recordings were 749 and 1062 epochs long, respectively. Of the scored epochs, 18.4 per cent were Wake, 7.1 per cent were N1, 48.1 per cent were N2, 10.1 per cent were N3, and 15.6 per cent were REM.

The OA between Waveband and the consensus of human scorers over the 36 474 epochs was 85.6 per cent [95% CI = 83.9%, 87.3%]. Table 3 reports positive and negative percent agreements and their CIs. The highest positive percent agreements were for N3 (98.2 per cent) and REM (91.6 per cent), while the lowest was for N1 (57.9 per cent). Negative percent agreement was above 93 per cent for all sleep stages, with the lowest negative percentage agreements being for N2 and N3, at 93.9 per cent and 93.4 per cent, respectively.

**Table 3:** Positive and negative percent agreement for each of the five sleep stages. We report the average positive percent agreement and negative percent agreement across the 38 subjects and the 95 per cent confidence intervals (CIs) in brackets. CIs are computed using bootstrap over the 38 subjects. For each sleep stage, the total number of epochs is shown.

Stage	Consensus epochs	Positive percent agreement	Negative percent agreement
*Wake*	6719	88.5 [85.3, 91.4]	98.0 [97.1, 98.7]
*N1*	2582	57.9 [53.0, 62.8]	98.0 [97.6, 98.3]
*N2*	17 534	83.4 [80.7, 85.7]	93.9 [92.3, 95.3]
*N3*	3922	98.2 [96.6, 99.4]	93.4 [91.9, 94.9]
*REM*	5690	91.6 [86.0, 95.8]	97.3 [96.2, 98.3]

**Figure 4:**
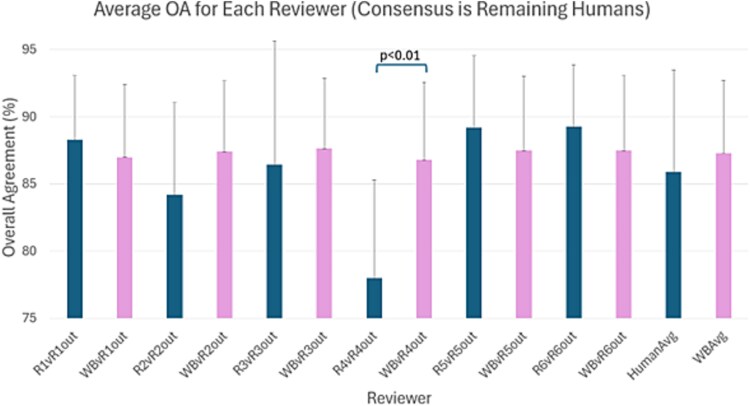
OA percent, averaged across 38 PSGs, of each human and Waveband versus the consensus opinion of the remaining 5 humans. For example, the OA for reviewer 1 (R1vR1out) is the average OA across all studies as compared to the consensus of reviewers 2 through 6 (left R1 out). WVvR1 is the average OA of Waveband across all studies as compared to the consensus of reviewers 2 through 6. Human and Waveband overall averages are shown in the last columns. Error bars indicate one *SD*. Only R4v4 was significantly different (*p* < .01) than Waveband performance after Bonferroni correction.

To better understand the disagreement between Waveband and the consensus of scorers, we present the confusion matrix in Table 4. Most of the errors made by Waveband occurred on epochs scored as N1 or N3 by the consensus PSG. Specifically, N1 was mostly confused for Wake, N2, or REM sleep, while N2 was mostly confused for N3 by Waveband. In post-hoc analyses, we obtained sleep scoring from three additional reviewers (for a total of six human scorers for each PSG) and calculated a leave-one-out consensus based on the majority opinion of the remaining five reviewers. Less than 1.5 per cent of epochs were rejected due to no consensus (see Supplementary Figure 1). Waveband performance was compared to each human as measured against the leave-one-out consensus (e.g. Waveband and human 1 scores were compared to the consensus opinion of reviewers 2–6). This analysis was performed across all studies for each of the six human reviewer-consensus pairs. OA for each human and Waveband vs the leave-one-out consensus is shown in Figure 4. Human reviewer 4 had significantly worse OA than Waveband (*p* < .01 with Bonferroni correction) as compared to Waveband, but there was no statistically significant difference for any other human vs Waveband pair or between the average of all human vs Waveband performance. Waveband demonstrated an overall average OA as compared to the average leave-one-out human consensus score of 87.3 +/− 5.4 per cent. The average human OA, as compared to the leave-one-out human consensus scores, was 85.9 +/− 7.6 per cent (last column of Figure 4).

**Table 4:** Confusion matrix comparing Waveband and consensus of expert-scored PSG for all sleep epochs is summarized as percentages. For each sleep stage determined by the consensus of human experts (manual staging), the corresponding stage as determined by automated analysis of the Waveband device (automated analysis) is shown as a percentage of all such stages. For example, 90.70% of stages classified as Wake (W) by manual staging were also classified as W by automated analysis, while 4.40% of these stages were classified as N1 by automated analysis.

		Consensus from manual staging				
		W	N1	N2	N3	REM
**Waveband**	W	90.70%	14.10%	0.60%	0.10%	1.40%
**(Automated analysis)**	N1	4.40%	55.20%	1.80%	0.00%	1.00%
	N2	0.80%	21.80%	83.70%	2.10%	6.70%
	N3	0.20%	0.20%	11.70%	97.80%	0.20%
	REM	3.80%	8.70%	2.20%	0.00%	90.70%
	Unassigned	0.01%	0.00%	0.00%	0.00%	0.00%
	Epoch count	6719	2582	17534	3922	5690

**Figure 5:**
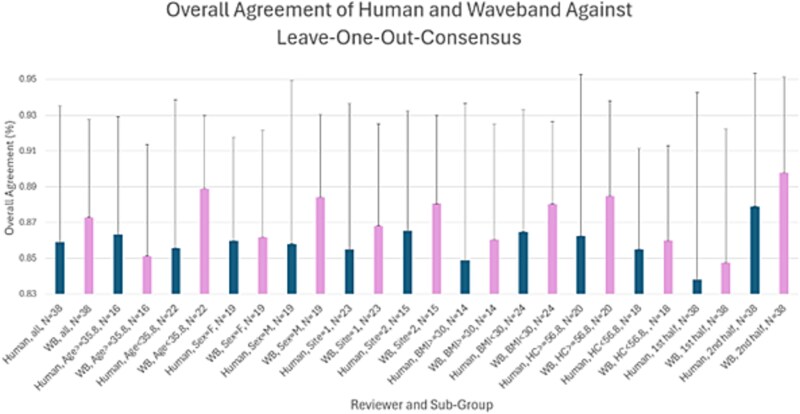
OA percent, averaged across sub-groups including those with BMI less than vs greater than 30, HC greater than or less than 56.8 cm, those older or younger than 36.8 years, males vs females, site 1 (United States) vs site 2 (France), and first vs second half of the night. Error bars indicate one *SD*. See Supplementary Table 1 for *p*-values, but in summary, Waveband age greater than mean and Waveband age less than mean, human age greater than mean and Waveband age greater than mean, and both human and Waveband first vs second half of the night comparisons were statistically significant after Bonferroni correction.

We then examined the performance of Waveband and humans for specific sub-groups (note that the study was not powered or designed for these sub-group analyses, and no effort was made to determine interactions between sub-group classes). Specifically, we examined groupings based on high vs low BMI (with 30 as the cutoff), high vs low head circumference (HC) (with 56.8 cm, the average of our population, as the cutoff), female vs male, age greater or less than mean (35.8 years), site (United States vs France), and first vs second half of the night. Results are shown in Figure 5, which also includes overall average performance (first two bars). Waveband showed slightly superior OA in all subgroupings except age >35.8 years. However, most of these pairings showed no Bonferroni-corrected statistical difference between humans and Waveband, except for age <35.8 years (humans superior), first half of the night (Waveband superior), and second half of the night (Waveband superior). Also, both Waveband and humans showed significantly different performance characteristics for the first vs second half of the night. Supplementary Table 1 shows the statistical comparisons and *p*-values. Despite these statistical differences, the actual magnitude of all differences was generally <2 per cent (note the scale bar of the ordinate in Figures 4 and 5), except for Waveband OA for the first half of the night as compared to Waveband OA for the second half of the night, which demonstrated a 5 per cent difference in favor of the second half of the night (*p* < .01). Nevertheless, Waveband performance for both halves of the night was still significantly superior to average human performance.

Note that the above analyses were performed after excluding three nights of poor-quality Waveband data. The primary reason for this poor quality was poor fitment, usually due to improper band size adjustment. To assess the impact of excluding these recordings, we also performed a worst-case OA analysis in which we included the three previously excluded Waveband recordings with the following assumptions:

1. For each of the recordings, Waveband sleep staging would have a 0 per cent agreement with every human’s PSG staging (worst-case); and,
2. For each of the recordings, human PSG sleep staging would have that human’s average OA versus the remaining leave-one-out consensus (e.g. we assumed reviewer 1 would have 88 per cent OA with the consensus of reviewers 2 through 6).
3. One PSG recording was excluded, but we did not include this as a failure (it remained excluded, in the spirit of a worst case for Waveband analysis).

As expected, this reduced the aggregate performance of Waveband (because we assume that Waveband would have had 0 per cent OA), while the average OA of human reviewers was unchanged (because we assumed their performance would be at their average). Specifically, Waveband average OA fell to 80.9 per cent, while human OA remained at 85.9 per cent (see Supplementary Figure 2). In this worst-case analysis, Waveband only outperformed one human expert. Although the *p*-value must be interpreted cautiously (because our assumptions reduced human variability and increased Waveband variability), there was still no statistically significant difference between Waveband and human expert performance. However, in real-world use, such poor recordings would be identified immediately following recording, allowing for repeat study with corrected fitment. This was done in the outpatient usability and data quality evaluation of Waveband, as reported separately in the LIVIE studies (see Savietto *et al.* 2026 [28]).

**Figure 6:**
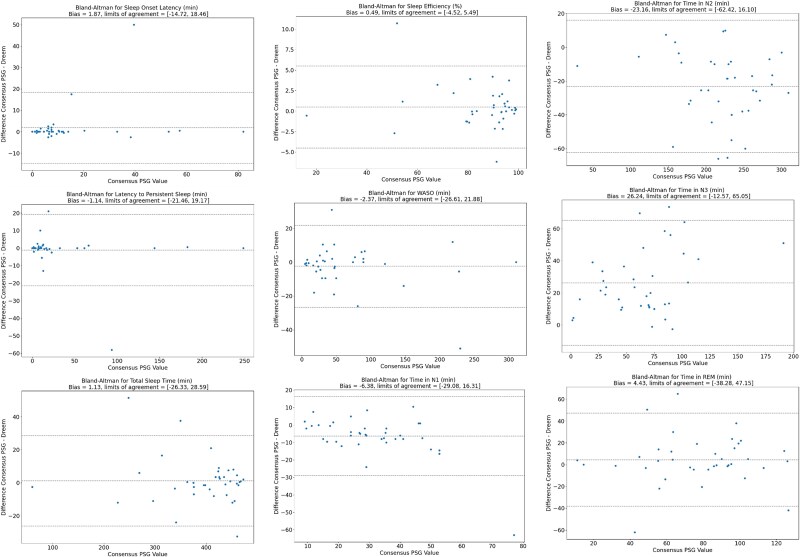
A series of Bland–Altman plots were generated to compare the estimates of sleep parameters obtained by the Waveband device and those obtained by the consensus of scorers on the PSG data. The x-axis of each plot represents the value estimated by the consensus of scorers, while the y-axis represents the difference between this value and the estimation provided by Waveband. Dashed lines indicate bias and limits of agreement (two *SD*). The plots were computed on the set of evaluable records, comprising 38 recordings, and each recording was represented by a data point.

**Table 5:** Average value and standard deviation for each of the sleep parameters (including TST, SE, etc.) are shown as estimated by Waveband and the consensus of scorers over the evaluable population (*n* = 38). The ICC is computed between the estimated value by Waveband and the consensus on PSG data over the evaluable population (*n* = 38). 95 per cent bootstrapped CIs are reported between brackets

Parameter	Mean ± SD		ICC [95% CI]
	PSG consensus	Waveband	
TST	391.2 ± 85.8	392.3 ± 83.5	0.99 [0.98; 0.99]
*SE*	84.8 ± 17.3	85.3 ± 16.8	0.99 [0.98; 0.99]
*LPS*	32.4 ± 54.3	31.3 ± 52.5	0.98 [0.96; 0.99]
*SOL*	13.1 ± 17.4	15.0 ± 19.2	0.89 [0.80; 0.94]
*WASO*	68.3 ± 73.1	65.9 ± 70.3	0.98 [0.96; 0.99]
*Time in sleep stage N1*	34.0 ± 18.6	27.6 ± 12.3	0.66 [0.44; 0.81]
*Time in sleep stage N2*	230.7 ± 57.6	207.6 ± 54.5	0.86 [0.75; 0.92]
*Time in sleep stage N3*	51.6 ± 33.7	77.8 ± 40.6	0.65 [0.43; 0.80]
*Time in REM stage*	74.9 ± 30.4	79.3 ± 30.6	0.73 [0.54; 0.85]

### Estimation of sleep parameters

Sleep parameters provide an overview of the night’s sleep architecture. For each sleep parameter, we calculated the average for Waveband and the consensus PSG and ICCs between the Waveband and consensus PSG estimation (Table 5). ICCs were above 0.95 for TST, SE, LPS, and WASO, but the time spent in N1, N3, and REM had the lowest ICCs, with values of 0.66, 0.65, and 0.73, respectively. Waveband appears to moderately overestimate the time in N3 and underestimate the time spent in N2. Bland–Altman plots (Figure 6) show that the difference between PSG and Waveband is centered around zero for most sleep parameters except N2 and N3. Time in N2 is underestimated, while time in N3 is overestimated by Waveband.

## Discussion

The Waveband sleep headband demonstrates high accuracy in sleep staging with an OA of 85.6 per cent versus a human consensus (surpassing the OA of 82 per cent for human expert inter-rater agreement). When recalculated using 6 human reviewers and using a leave-one-out consensus, the aggregate OA for Waveband was 87.3 per cent, and the average human consensus was 85.9 per cent (not statistically significantly different). This agreement is also comparable, or superior to previously reported inter-rater agreement among human experts scoring PSG data from healthy young adults [18], patients with sleep apnea [26], and adult women [29]. Although demographic differences exist among these studies, the results suggest that the Waveband performs comparably to a trained sleep technologist scoring data acquired by a full PSG montage, despite electrode positions that do not match typical PSG EEG electrodes (which has been confirmed by prior studies of just frontopolar EEG locations demonstrating good staging correlation to PSG-based staging [23]).

On post hoc subgroup analyses, human versus Waveband groupings did not show large differences in OA, although Waveband consistently demonstrated slightly superior OA except for younger age (the only grouping in which Waveband performed worse than humans was in subjects younger than 35.8 years). However, the differences between expert humans and Waveband were small, with both sets of reviewers performing well. Nevertheless, some notable differences were found. Performance for the first versus second half of the night was the largest difference, with Waveband performing significantly better on the second half of the night (which could reflect the lower percentage of N1 and N3 during these later epochs, and these stages were identified as the hardest to classify). This trend was also true for human reviewers (performing worse in the first half of the night), and human performance was statistically significantly lower than Waveband performance for both first and second half of the night. Note that this study was not powered or designed for these sub-groupings, and we did not attempt to correct for covariates among the subgroups.

Performance evaluation for sleep metrics demonstrated ICCs exceeding 0.9 for the Waveband device as compared to human review of PSG, with excellent agreement for estimating TST, SE, LPS, and WASO. Waveband provided a good estimation of SOL, with an agreement above 0.75. Kuna *et al.* [30] analyzed ICCs between different sleep centers for TST and SE and found average ICCs between centers to be 0.87 [0.85, 0.89] for TST and 0.77 [0.73,0.80] for SE, both lower than the ICCs observed in the present study between Waveband and the consensus scoring. As TST, SOL, SE, LPS, and WASO are critical for assessing and measuring sleep disruption and objectively evaluating insomnia symptoms, the high accuracy observed with the Waveband makes it a useful tool for diagnosing and managing disordered sleep at home without the need for technologist intervention.

Agreement for individual sleep stage labels was high for time spent in N2 (0.86 CI = [0.75; 0.92]) and moderate for time spent in N1, N3, and REM (0.66 CI = [0.44; 0.81], 0.65 CI: [0.43; 0.80], 0.73 CI = [0.54; 0.85], respectively). Kuna *et al.* reported similar (albeit lower) agreement values between human experts at different sleep centers for estimating the duration of N1 (0.44 CI = [0.39,0.49]), N2 (0.61 CI = [0.57, 0.66]), N3 (0.40 CI = [0.35, 0.45]), and REM (0.685 CI = [0.64, 0.72]). The agreement between the Waveband and consensus PSG scoring falls within the range of inter-rater human variability typically observed in sleep studies. This was true even when including three studies that were excluded in the original analysis due to poor Waveband quality—which in real-world practice could be corrected with a repeat study, given the ease of performing multi-night recording (as we demonstrated in the outpatient LIVIE studies, reported in Savietto *et al.* 2026 [28]). The Waveband device does have on-board data quality assessment to flag low quality recordings—but this feature was not available at the start of this study and therefore not evaluated here. However, it is noteworthy that Waveband tends to overscore N3 sleep, which could introduce a bias when comparing it to other devices. This bias may be due to a lower threshold for Waveband to score slow-wave sleep instead of N2 and could be corrected in future sleep staging model revisions using existing data.

Several devices are available for at-home EEG measurements to assess sleep in subjects. In Younes *et al.* [31], ICCs reported for TST, SE, SOL, N1, N2, N3, and REM were 0.86, 0.60, 0.31, 0.88, 0.57, 0.86, 0.58, and 0.83, respectively, between the Prodigy Sleep Monitor (Cerebra Health Inc., Winnipeg, Canada) and human scorers, below the values we report for Waveband in Table 5. The Self-Applied Somnography (SAS) (Nox Medical, Reykjavik, Iceland) is a self-applicable, simplified EEG montage, developed for at-home research use. Automated sleep staging evaluated on the SAS [32] reported accuracy ranging from 72.3 to 80.5 per cent depending on the montage on subjects with suspected bruxism or apnea (average Apnea Hypopnea Index (AHI): 12.7–15.7). In a study conducted with the Sleep Profiler (Advanced Brain Monitoring, Carlsbad, California, United States), which uses EEG frontal derivations, Levendowski *et al.* [23] showed that clinicians were able to assess sleep over two consecutive nights in a clinical population (diagnosed Insomnia/depression, or comorbid Insomnia and sleep apnea or all three), demonstrating strong agreement between PSG and the Sleep Profiler. These authors showed a clinical benefit of averaging two nights to improve the assessment of sleep abnormalities and the balance of sleep architecture and sleep continuity biomarkers. The Sleep Profiler [19] reported an OA of 71.3 per cent between automated sleep staging (without additional sleep technologist editing) and consensus scoring. Despite an overall good agreement with PSG, most of the predecessor devices show lower performance in wake detection and sleep stage transitions.

Additionally, all the above devices use wet and disposable EEG electrodes, which require a longer time for subject set-up and require manually placed EEG sensors. Dry EEG electrodes offer the advantage of minimal setup (placement is achieved with only proper positioning of the headband), easily repeated usage, and occipital EEG coverage (where self-applied stickers would fall into the hairline for many patients)—but at the cost of higher artifact than wet EEG. Nevertheless, the Waveband dry sensor EEG system demonstrated excellent agreement to in-lab PSG using standard wet electrodes. Waveband can be set up quickly, has no consumable parts, and allows multiple nights of data collection from the subject’s home. Repeated use is limited only by subject adherence, as the device is designed for durability and reuse. Waveband appears to perform well compared to the three devices discussed and in-lab PSG.

Several limitations impact this study. Here, we focused on participants with chronic insomnia, and therefore, we are only able to hypothesize that our results would generalize to a broader sleep clinic population with more diverse sleep pathology. Furthermore, the current study did not characterize the impact of insomnia severity on staging, which could impact generalization to milder or more severe insomnia patients. While insomnia severity and additional sleep pathology do not alter the underlying structure of sleep architecture used to stage sleep, further investigation would need to address the utility of Waveband in other populations. Performance was not worse than expert human performance when assessing impacts of age, HC, BMI, sex, or clinical site (United States or France).

Our results demonstrate that Waveband’s automatic staging and output are comparable with human expert inter-scorer variability and outperform published results from similar devices. A self-applied, EEG-based sleep assessment device, with an accuracy comparable to laboratory PSG, and which can be used over multiple nights, could provide a valuable additional tool for the assessment and management of patients with disturbed sleep. Waveband might therefore become part of clinical pathways as an alternative to sleep diaries and actigraphy, providing more accurate and objective assessments to inform management, without the practical difficulties of obtaining longitudinal in-lab PSG studies. In this study, a population with chronic insomnia was chosen, given the need for accurate sleep staging in this population and because this condition reflects a common and underdiagnosed neuropsychiatric condition that might benefit from improved monitoring. Longitudinal sleep monitoring in the home setting may allow for improved diagnosis and targeted treatments.

## Conclusions

The Waveband is a wearable EEG device that is self-applied and was recently FDA cleared for use in a home or healthcare setting. Currently, technologies are underutilized for diagnosing and assessing insomnia due to the low specificity of many actigraphy devices used in a home setting for differentiating sleep from wakefulness. This can result in overestimation of TST and underestimation of WASO time [33]. While PSG is accurate, the sleep lab environment does not allow for repeated measures of sleep and can impact patients’ ecological sleep [34]. Similarly, ambulatory PSG devices are not used for multiple measurements due to the need for technical set-up.

Overall, these results suggest that Waveband addresses the unmet need for more accurate sleep staging in a home setting. Waveband could therefore be used as a clinical tool for assessing sleep in patients with insomnia symptoms and disturbed sleep, where more accurate sleep staging is required than what can be provided by actigraphy or existing home sleep tests, where additional sensors are not indicated, and where in-lab sleep assessment is not available or indicated. Its design also allows for longitudinal use, enabling Waveband to assess night-to-night variability in sleep or how sleep changes over time in the patient’s own home. The characteristics and capabilities of this system also make it an attractive tool for sleep assessments in clinical trials. Future studies will be needed to explore generalizability beyond insomnia, but we believe that this study contributes to the development of simpler and more accurate methods for objectively assessing sleep quality and variability in patients with disturbed sleep.

## Supplementary Material

zsag069_Waveband_Octave_3_Sleep_REV5_3_PRINT_SUPPLEMENTARY_MATERIALS_CLEAN

## Data use policy

The data from this trial are proprietary and unable to be shared.
