# Supplementary material for: Multicenter evaluation of the Waveband system for automated sleep assessment in patients with insomnia symptoms
Source: Sleep. 2026 Mar 28;49(7):zsag069. doi: 10.1093/sleep/zsag069 (PMC13357494; doi:10.1093/sleep/zsag069)
Supplement: zsag069_Waveband_Octave_3_Sleep_REV5_3_PRINT_SUPPLEMENTARY_MATERIALS_CLEAN [file zsag069_waveband_octave_3_sleep_rev5_3_print_supplementary_materials_clean.docx]

**Multicenter evaluation of the Waveband System for automated sleep assessment in patients with** **insomnia symptoms**

## **Authors and Institutions**

Silvia Frati Savietto [Beacon Biosignals], Antoine Guillot [Beacon Biosignals], Mason Harris [Beacon Biosignals], Jay Pathmanathan [Beacon Biosignals], Alexander M. Chan [Beacon Biosignals], M. Brandon Westover [Beacon Biosignals], Derek Hill [Panoramic Digital Health], Valérie Bertaina-Anglade [Biotrial Neurosciences, Rennes (FR)], Geoffrey Viardot [Biotrial Neurosciences, Rennes (FR)], Pierrick Arnal [Dreem SAS], and Jacob Donoghue [Beacon Biosignals].

### **Corresponding author's name, address and, valid email address**

Frati Savietto Silvia

- silvia.fratisavietto@beacon.bio
- 22 Boston Wharf Rd, Boston, MA 02210, United States

##

## **Appendix**

**Definitions**^36^**:**

- Positive percent agreement (new/non ref. std.) — proportion of non-reference standard positive subjects in whom the new test is positive
- Negative percent agreement (new/non ref. std.) — proportion of non-reference standard negative subjects in whom the new test is negative
- Overall agreement — proportion of subjects in whom the new test and the non-reference standard give the same outcome
- Intraclass Correlation Coefficient (ICC) — measure of the correlation between individuals clustered within the same context

*Supplemental Figure 1. Percent of sleep epochs (averaged across all 38 studies) that were rejected in each consensus. Consensus is indicated for the left out human reviewer (for example Consensus_R1 is the consensus opinion of reviewers 2 through 6). Error bars are one standard deviation. Averaged across all consensus scorers, an average of 1.3% of epochs were rejected.*

*Supplemental Figure 2. Worst-case overall agreement analysis. We repeated the analysis shown in Figure 4, but added back 3 nights of data that were previously excluded due to low Waveband quality. To complete this analysis, we assumed Waveband recordings would have 0% agreement with human graded PSG (the worst case), while humans performed at their mean OA as compared to the consensus of all other humans. Here, average Waveband performance is lower, but still not statistically different from human experts (none of the Bonferroni corrected comparisons was statistically significant at p<0.05).*

| Group | p | Rater with higher OA |
| --- | --- | --- |
| All studies | ns | WB superior |
| Age, human rating age>=35.8y vs human rating age<35.8y | ns | human rating age>=35.8y superior, 86.3% vs 85.6% |
| Age, WB rating age>=35.8y vs WB rating age<35.8y | <0.01 | WB rating age<35.8y superior, 88.9% vs 85.1% |
| Age, human rating age>=35.8y vs WB rating age>=35.8y | <0.01 | human rating age>=35.8y superior, 86.3% vs 85.1% |
| Age, human rating age<35.8y vs WB rating age<35.8y | ns | WB rating age<35.8y superior, 88.9% vs 85.6% |
| Sex, human rating F vs human rating M | ns | human rating F superior, 86% vs 85.8% |
| Sex, WB rating F vs WB rating M | ns | WB rating M superior, 88.4% vs 86.2% |
| Sex, human rating F vs WB rating F | ns | WB rating F superior, 86.2% vs 86% |
| Sex, human rating M vs WB rating M | ns | WB rating M superior, 88.4% vs 85.8% |
| Site, human rating 1 vs human rating 2 | ns | human rating 2 superior, 86.5% vs 85.5% |
| Site, WB rating 1 vs WB rating 2 | ns | WB rating 2 superior, 88.1% vs 86.8% |
| Site, human rating 1 vs WB rating 1 | ns | WB rating 1 superior, 86.8% vs 85.5% |
| Site, human rating 2 vs WB rating 2 | ns | WB rating 2 superior, 88.1% vs 86.5% |
| BMI, human rating BMI>=30 vs human rating BMI<30 | ns | human rating BMI<30 superior, 86.5% vs 84.9% |
| BMI, WB rating BMI>=30 vs WB rating BMI<30 | ns | WB rating BMI<30 superior, 88% vs 86% |
| BMI, human rating BMI>=30 vs WB rating BMI>=30 | ns | WB rating BMI>=30 superior, 86% vs 84.9% |
| BMI, human rating BMI<30 vs WB rating BMI<30 | ns | WB rating BMI<30 superior, 88% vs 86.5% |
| HC, human rating HC>=56.8 vs human rating HC<56.8 | ns | human rating HC<56.8 superior, 86.5% vs 84.9% |
| HC, WB rating HC>=56.8 vs WB rating HC<56.8 | ns | WB rating HC<56.8 superior, 88% vs 86% |
| HC, human rating HC>=56.8 vs WB rating HC>=56.8 | ns | WB rating HC>=56.8 superior, 86% vs 84.9% |
| HC, human rating HC<56.8 vs WB rating HC<56.8 | ns | WB rating HC<56.8 superior, 88% vs 86.5% |
| Site, human rating 1 vs human rating 2 | ns | human rating 2 superior, 86.5% vs 85.5% |
| Site, WB rating 1 vs WB rating 2 | ns | WB rating 2 superior, 88.1% vs 86.8% |
| Site, human rating 1 vs WB rating 1 | ns | WB rating 1 superior, 86.8% vs 85.5% |
| Site, human rating 2 vs WB rating 2 | ns | WB rating 2 superior, 88.1% vs 86.5% |
| Half of night, human rating 1st half vs human rating second half | <0.01 | human rating second half superior, 87.9% vs 83.8% |
| Half of night, WB rating first half vs WB rating second half | <0.01 | WB rating second half superior, 89.8% vs 84.8% |
| Half of night, human rating first half vs WB rating first half | <0.01 | WB rating first half superior, 84.8% vs 83.8% |
| Half of night, human rating second half vs WB rating second half | <0.01 | WB rating second half superior, 89.8% vs 87.9% |

### Supplementary Table 1. Group wise comparisons performed. Ns = non significant (all p-values are Bonferroni corrected for the 29 listed comparisons). For each grouping, the rater with the higher OA is listed. WB = Waveband.
